# Deubiquitinase USP7 regulates *Drosophila* aging through ubiquitination and autophagy

**DOI:** 10.18632/aging.104067

**Published:** 2020-11-20

**Authors:** Lang Cui, Wenhao Song, Yao Zeng, Qi Wu, Ziqiang Fan, Tiantian Huang, Bo Zeng, Mingwang Zhang, Qingyong Ni, Yan Li, Tao Wang, Diyan Li, Xueping Mao, Ting Lian, Deying Yang, Mingyao Yang, Xiaolan Fan

**Affiliations:** 1 Institute of Animal Genetics and Breeding, Sichuan Agricultural University, Chengdu, Sichuan, P. R. China; 2 Farm Animal Genetic Resources Exploration and Innovation Key Laboratory of Sichuan Province, Sichuan Agricultural University, Chengdu, Sichuan, China

**Keywords:** USP7, *Drosophila*, aging, autophagy, DMC

## Abstract

Ubiquitination-mediated protein degradation is the selective degradation of diverse forms of damaged proteins that are tagged with ubiquitin, while deubiquitinating enzymes reverse ubiquitination-mediated protein degradation by removing the ubiquitin chain from the target protein. The interactions of ubiquitinating and deubiquitinating enzymes are required to maintain protein homeostasis. The ubiquitin-specific protease USP7 is a deubiquitinating enzyme that indirectly plays a role in repairing DNA damage and development. However, the mechanism of its participation in aging has not been fully explored. Regarding this issue, we found that USP7 was necessary to maintain the normal lifespan of *Drosophila melanogaster*, and knockdown of *dusp7* shortened the lifespan and reduced the ability of *Drosophila* to cope with starvation, oxidative stress and heat stress. Furthermore, we showed that the ability of USP7 to regulate aging depends on the autophagy and ubiquitin signaling pathways. Furthermore, 2,5-dimethyl-celecoxib (DMC), a derivative of celecoxib, can partially restore the shortened lifespan and aberrant phenotypes caused by *dusp7* knockdown. Our results suggest that USP7 is an important factor involved in the regulation of aging, and related components in this regulatory pathway may become new targets for anti-aging treatments.

## INTRODUCTION

Aging is a natural event, driven by multiple genetic and environmental factors and is also a gradual and irreversible process [1, 2]. Research on aging has made great progress. The reasons for aging are diverse, and their typical hallmarks are described as follows: genomic instability, telomere attrition, epigenetic alterations, loss of proteostasis, deregulated nutrient sensing, mitochondrial dysfunction, cell senescence, stem cell failure and altered intercellular communication [3, 4].

Proteostasis is maintained by a proteostasis network [5]. This network, which can sense and respond to misfolded proteins in all cells individually or together in different subnetworks, is composed of molecular chaperones, protein degradation mechanisms, and stress response pathways [6]. However, during aging, the ability of cells to maintain protein stability is weakened [7]. The molecular chaperone-mediated degradation of protein equilibrium mechanisms is disrupted, leading to increased protein oxidation, mis-location and aggregation in organisms [8]. The misfolded proteins cannot be degraded, and they accumulate in the body, eventually causing aging or aging-related diseases (such as Alzheimer's disease (AD) and Parkinson’s disease (PD)) in humans [9].

Protein is degraded via two primary pathways: the ubiquitin-proteasome pathway (UPS) and the autophagic lysosomal pathway [10]. According to the occurrence of autophagy, it includes three forms: macroautophagy, microautophagy, and molecular chaperone-mediated autophagy [11]. The main role of autophagy is to degrade many different substrates during metabolic stress to maintain the energy balance [12], and it is usually used to degrade cytoplasmic components, such as organelles and macromolecular compounds [13]. Lysosomes are an essential catalytic part of the autophagic lysosomal degradation system [14]. The lysosome contains a large number of hydrolases that are involved in the degradation of proteins, which are decomposed into small peptides and amino acids to obtain energy or synthesize new proteins [15]. The autophagic lysosomal pathway maintains protein homeostasis through this mechanism of degradation.

The ubiquitin-proteasome pathway is the major selective protein degradation pathway in eukaryotic cells [13]. The UPS can mediate the degradation of short-lived regulatory proteins, ensuring the orderly progression of related biological processes and promoting the degradation of defective proteins [10, 16]. The ubiquitin-proteasome pathway consists of a protein recruiting pathway and a protein degradation pathway. The first part consists of three enzymes: ubiquitin-activating enzyme (E1), ubiquitin-conjugation enzymes (E2), and ubiquitin ligase enzymes (E3). As a result of the cascade of three ubiquitinases, ubiquitin eventually binds to the substrate protein. Protein degradation is mainly accomplished by the proteasome, where the substrate protein is recognized and degraded by the proteasome [5].

Before the ubiquitin cascade forms a sufficiently large branched structure to activate the proteasome, the deubiquitinating enzymes (DUBs) can remove the ubiquitin molecule, reversing the ubiquitination process and maintaining protein stability; this process protects the protein from degradation [17].

The *Drosophila* ubiquitin-specific protease 7 (dUSP7) is one of the most abundant deubiquitinating proteases. Many reports had proved that dUSP7 is closely related to various biological processes. dUSP7 plays a very important role in DNA repair, Tip60-mediated apoptosis, various cancers, and organism development [18–21]. Besides, previous studies have revealed that Usp7 is essential for some organs development and size, such as wing development [14], brain size [22], eye development [23].

Only a few studies on the role of dUSP7 in aging have been reported. In *C. elegans*, the lack of math-33, a homologous protein of dUSP7, can reduce the lifespan of *C. elegans* [24]. To explore whether the regulatory mechanism of dUSP7 in aging is conserved, we examined the aging effects of dUSP7 in *Drosophila.* As the signaling pathways that regulate aging are evolutionarily conserved, our results could further elucidate the mechanism of dUSP7 in the regulation of aging.

## RESULTS

### Knockdown of *dusp7* significantly shortens the *Drosophila* lifespan

**Figure 1:**
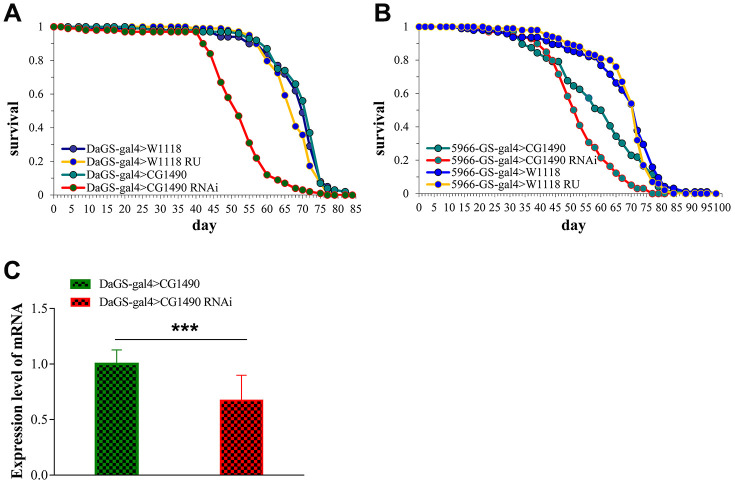
**The effect of *dusp7* knockdown on the lifespan of *Drosophila*.** (**A**) The *dusp7* expression level when RU486 was added to induce *dusp7* knockdown (*** *p*<0.001; > means hybridization). (**B**) The lifespan curve when *dusp7* was knocked down in *Drosophila*. (**C**) The lifespan curve under specific knockdown of *dusp7* in the *Drosophila* intestinal track.

Using classical Gal4 driver and GeneSwitch system of *Drosophila* to design a gene which is able to be time and tissue-specific expression (Figure 1A). CG1490 RNAi is with *usp7*-RNAi gene strains of *Drosophila*. To explore the effect of ubiquitin-specific protease dUSP7 on the lifespan of *Drosophila*, we first tested the mRNA levels of *dusp7* when *dusp7* was knocked down by *Da^GS^*-*gal4*. The results showed that the expression of *dusp7* was significantly decreased compared with the control group (Figure 1C). When *dusp7* was knocked down in *Drosophila*, the lifespan was significantly decreased in comparison with the control group. The decrease was up to approximately 28.9%, with the mean lifespan decreasing from 69.1 to 52.1 days, p<0.0001 (Figure 1A). When we knocked down the expression of *dusp7* only in *Drosophila* guts, the lifespan was also significantly decreased compared with the control group by up to approximately 13.3%, with the mean lifespan decreasing from 58.7 to 52.7 days, *p*<0.0001 (Figure 1B). These results indicate that dUSP7 is necessary to maintain the lifespan in *Drosophila*.

### Knockdown of *dusp7* significantly decreased the climbing ability of *Drosophila*

**Figure 2:**
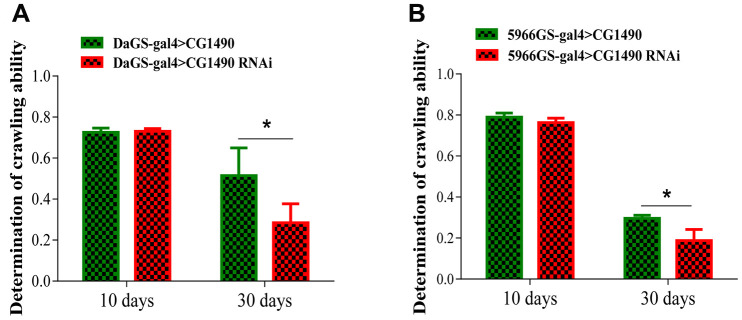
**Knockdown of *dusp7* decreases the climbing ability of *Drosophila*.** (**A**) Effect on the climbing ability of *Drosophila* after *dusp7* knockdown (* *p*<0.05). (**B**) Effect on the climbing ability of *Drosophila* after *dusp7* knockdown in the gut (* *p*<0.05). (>: hybridization).

The climbing ability of *Drosophila* weakens as the *Drosophila* age. To explore the climbing ability of *Drosophila* upon *dusp7* knockdown, we tested the climbing ability of 10-day-old and 30-day-old *Drosophila*. For the 10-day-old flies, the climbing ability did not change between the control and knockdown groups; however, for the 30-day-old flies, the climbing ability was significantly decreased compared with the control group. The climbing ability performance index (PI) decreased from 0.515 to 0.285, *p*<0.05 (Figure 2A). We also gut-specifically knocked down the *dusp7* gene in *Drosophila*, and the climbing ability was significantly decreased compared with the control group, decreasing the PI from 0.29 to 0.17, *p*<0.05 (Figure 2B). These findings indicate that *dusp7* can reduce the muscle mass and cause the flies to weaken, thereby decreasing the climbing ability.

### Knockdown of *dusp7* promotes gut dysfunction in *Drosophila*

**Figure 3:**
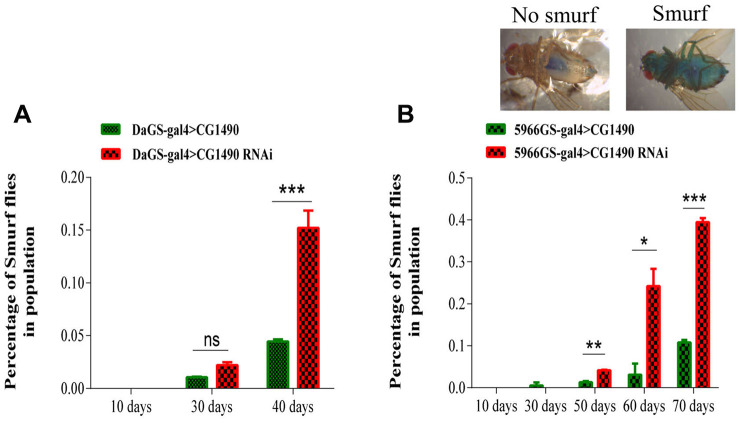
**Effect of *dusp7* knockdown on the intestinal function of *Drosophila*.** (**A**) Effect on the intestinal integrity of *Drosophila* after systemic knockdown of the ubiquitin-specific protease dUSP7(*** *p*<0.001). (**B**) Effect on intestinal integrity of *Drosophila* after gut-specific knockdown of the ubiquitin-specific protease dUSP7 (* *p*<0.05; ** *p*<0.01; *** *p*<0.001). (>: hybridization).

As gut function integrity is closely related to aging, it is important for the health of the organism. Therefore, we investigated whether *dusp7* acts on the gut health of *Drosophila*. From the results of the “smurf” assay [25], we found that systemic knockdown and gut-specific knockdown of *dusp7* significantly increased the proportion of “smurfs” in 40-day-old females compared with controls (*p*<0.05; Figure 3A, 3B), indicating that *dusp7* knockdown reduces the gut barrier function.

### dUSP7 is necessarily for the stress tolerance of *Drosophila*

**Figure 4:**
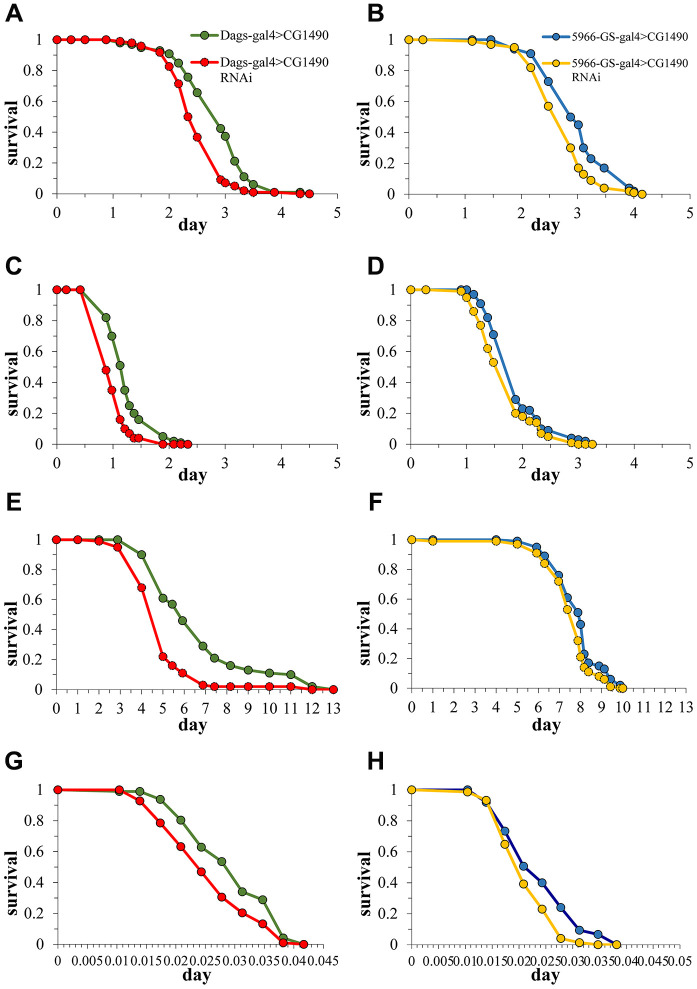
**Effect of *dusp7* knockdown on *Drosophila* stress tolerance.** (**A**) Effect of *dusp7* knockdown on *Drosophila* tolerance to H_2_O_2_. (**B**) Effect of gut-specific *dusp7* knockdown on *Drosophila* tolerance to H_2_O_2_. (**C**) Effect of *dusp7* knockdown on *Drosophila* tolerance to paraquat. (**D**) Effect of gut-specific *dusp7* knockdown on *Drosophila* tolerance to paraquat. (**E**) Effect of *dusp7* knockdown on *Drosophila* tolerance to starvation. (**F**) Effect of gut-specific *dusp7* knockdown on *Drosophila* tolerance to starvation. (**G**) Effect of *dusp7* knockdown on *Drosophila* tolerance to heat stimulation. (**H**) Effect of *dusp7* gut-specific knockdown on *Drosophila* tolerance to heat stimulation. (>: hybridization).

Because stress tolerance and longevity are mechanistically and phenotypically linked [26], we investigated changes in the stress tolerance of Drosophila by knocking down *dusp7*. The 10-day-old flies were used to examine susceptibility to starvation, heat-shock, paraquat, and H_2_O_2_ stress. The results showed that flies with *dusp7* knockdown (systemic or gut-specific) had significantly decreased tolerance to H_2_O_2_ compared with controls. The survival time for the systemic knockdown flies decreased by 23.08% (*p*<0.0001) and that of the gut-specific knockdown flies decreased by 10.5% (*p*<0.05) (Figure 4A, 4B). The tolerance to paraquat was similar to H_2_O_2_, where both systemic and gut-specific knockdown flies showed a significantly decreased the tolerance to paraquat compared with controls. The survival time for systemic knockdown decreased by up to 27.27% (*p*<0.0001), and gut-specific knockdown decreased by up to 10% (*p*<0.001) (Figure 4C, 4D). Moreover, we also validated the stress tolerance by using systemic overexpression (OE) *dusp7 Drosophila* (Supplementary Figure 1A)and the survival time showed a significantly increasing by up to 47.29%(*p*<0.0001) (Supplementary Figure 1B). Subsequently, we found that flies that were knocked down for *dusp7* showed significantly decreased tolerance to starvation compared with controls (the survival time for systemic knockdown decreased by up to 25.37%, *p*<0.0001, and gut-specific knockdown decreased by up to 3.79%, *p*<0.01, Figure 4E, 4F). We also tested for heat-shock tolerance, and the results were the same as above. Flies knocked down for *dusp7* (both systemic and gut-specific) showed significantly decreased tolerance to heat-shock compared with controls (the survival time for systemic knockdown decreased by up to 22.24%, *p*<0.001, and gut-specific knockdown decreased by up to 12.59%, *p*<0.01, Figure 4G, 4H). But there is no significant increasing in systemic overexpression *dusp7 Drosophila* (Supplementary Figure 1C). These results indicated that *usp7* is necessary for the stress tolerance of *Drosophila*.

### *dusp7* regulates protein degradation and autophagy

**Figure 5:**
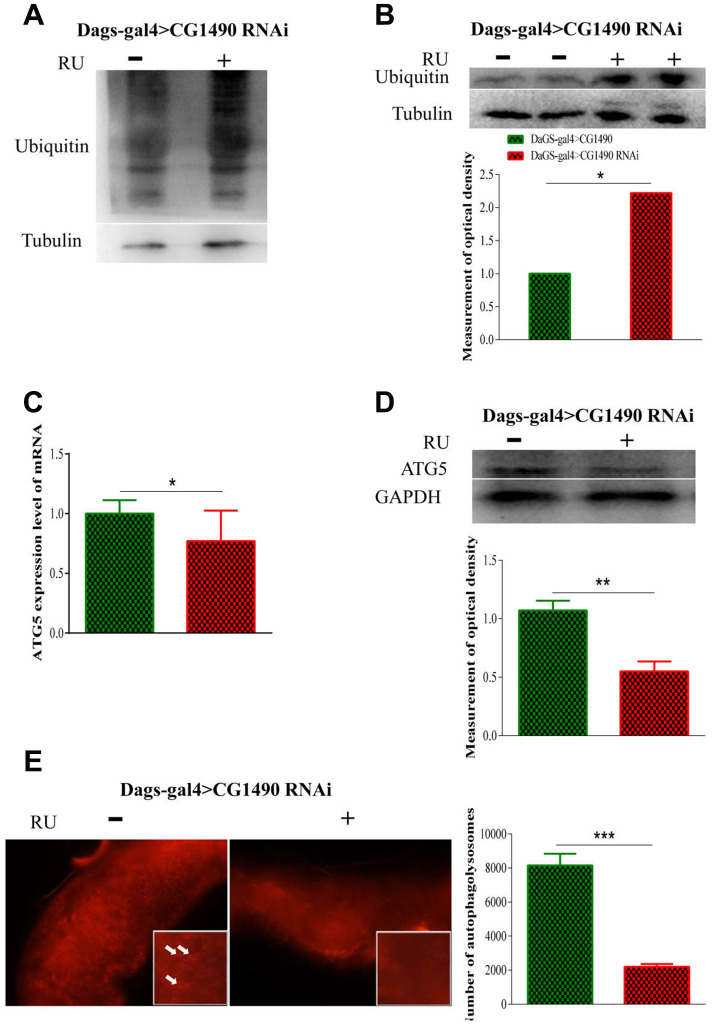
**Effect of *dusp7* in *Drosophila* on the control of protein in vivo.** (**A**) The protein ubiquitination level after *dusp7* knockdown in *Drosophila.* (**B**) The ubiquitination level after *dusp7* knockdown in *Drosophila* (* *p*<0.05)*.* (**C**) Autophagy-related gene expression after *dusp7* knockdown in *Drosophila* (* *p*<0.05). (**D**) Autophagy-related protein expression after *dusp7* knockdown in *Drosophila* (** *p*<0.01). (**E**) The effect of autolysosomes after *dusp7* knockdown in *Drosophila* (*** *p*<0.001; white arrow points to autophagosome). (>: hybridization; + means adding Ru to induce expression; - means no adding RU).

Loss of protein homeostasis is considered to be one of the "hallmarks of aging" [3, 27]. Protein homeostasis is maintained by the protein stabilization network (PN), which is composed of molecular chaperones, protein degradation mechanisms, and stress response pathways [6]. When *dusp7* was knocked down, we found that knockdown flies significantly increased their ubiquitination of proteins and monoubiquitin (Figure 5A, 5B). The *dusp7* knockdown flies also significantly decreased their protein and mRNA expression levels of autophagy-related 5 (atg5) (Figure 5C, 5D). Additionally, we detected a decrease of autolysosome in their midgut epithelial cells (Figure 5E). These results indicated that *dusp7* may regulate protein degradation and autophagy to some extent.

### DMC extends the lifespan of *dusp7-*knockdown drosophila

**Figure 6:**
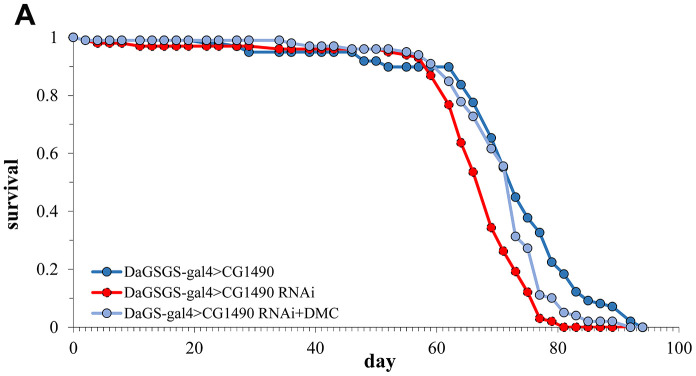
****Lifespan of *dusp7*-knockdown group after adding DMC (**A**) Effect on the lifespan of adding DMC to *dusp7*-knockdown *Drosophila* (>: hybridization).

2,5-Dimethyl celecoxib (DMC) is a derivative of celecoxib, a non-steroidal anti-inflammatory drug that inhibits colon cancer and induces apoptosis [28, 29]. We previously found that DMC can extend the lifespan of *Drosophila* by increasing autophagy in the cell [30]. Therefore, we tested whether the addition of DMC could rescue the shortened lifespan of *Drosophila* caused by the knockdown of *dusp7*. The results showed that DMC was able to rescue the shortened lifespan caused by *dusp7* knockdown (Figure 6A). Compared with the *dusp7-*knockdown group, the lifespan of the DMC-added group was significantly increased by approximately 6.36%, from 66 days to 70.2 days, *p*<0.0001.

### DMC restores the physiological indexes of *Drosophila* related to *dusp7* knockdown

**Figure 7:**
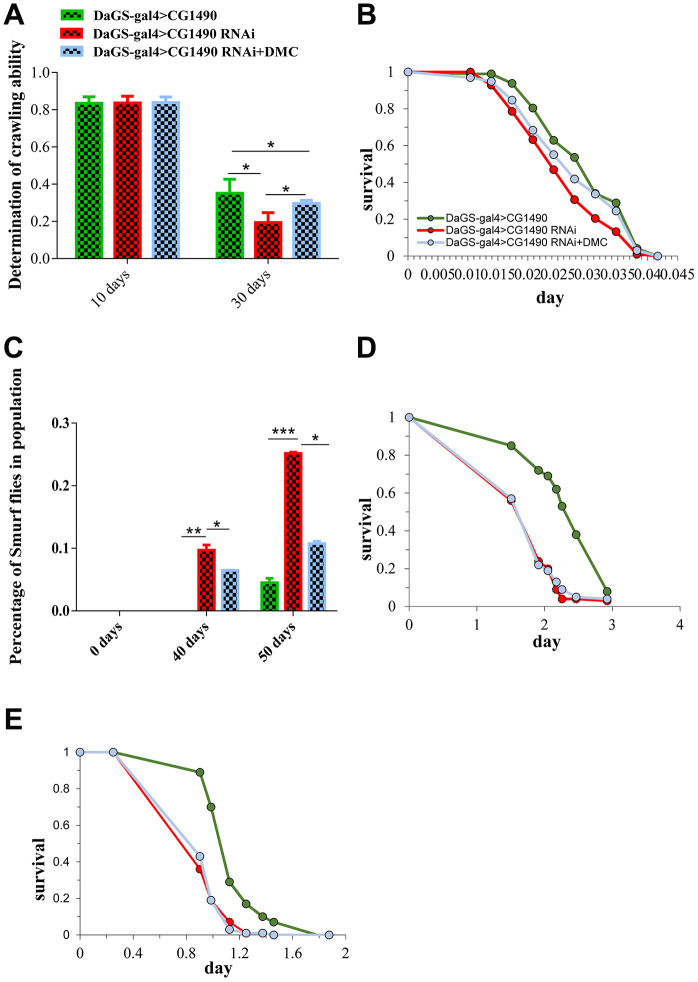
**Effect of DMC addition on the senescence traits of *dusp7-*knockdown *Drosophila*.** (**A**) Effect of DMC addition on *dusp7*-knockdown *Drosophila* (* *p*<0.05)*.* (**B**) Effect of DMC addition on heat-stimulation tolerance in *dusp7*-knockdown *Drosophila.* (**C**) Effect of DMC addition on H_2_O_2_ tolerance in *dusp7-*knockdown *Drosophila* (* *p*<0.05; ** *p*<0.01; *** *p*<0.001). (**D**, **E**) Effect of DMC addition on paraquat (**D**) and H_2_O_2_ (**E**) tolerance in *dusp7-*knockdown *Drosophila* *.* (>: hybridization).

An extended lifespan is often accompanied by the improvement of physiological indicators. Therefore, we examined the effects of DMC on physiological indicators such as climbing ability, antioxidant capacity and starvation resistance in *Drosophila*. We compared three groups of flies: non-knockdown, *dusp7* knockdown and knockdown plus DMC. We found that the climbing ability of flies in three group did not change for 10-day-old flies, but in 30-day-old flies, the climbing ability of the *dusp7*-knockdown group was significantly decreased compared with the control group; the climbing ability PI was decreased from 0.3508 to 0.1938 (*p*<0.05). The addition of DMC could significantly recover the climbing ability compared with the *dusp7-*knockdown group; the climbing ability PI was increased from 0.1938 to 0.2959 (*p*<0.05) (Figure 7A). Next, we tested the tolerance to heat. The results showed that the mean lifespan in the *dusp7*-knockdown group was significantly decreased compared with the control group; the survival time decreased by up to 12.6% (*p*<0.001). The addition of DMC significantly increased the mean lifespan compared with the *dusp7*-knockdown group; the survival time increased by up to 7.5% (*p*<0.05) (Figure 7B). Also the DMC added can partly restore the phenotype of intestinal barrier integrity disorder caused by usp7 knockdown (Figure 7C). However, DMC could not restore the antioxidant capacity in the *dusp7*-knockdown group (Figure 7D, 7E).

### DMC improves autophagy and ubiquitination in *dusp7-*knockdown *Drosophila*

**Figure 8:**
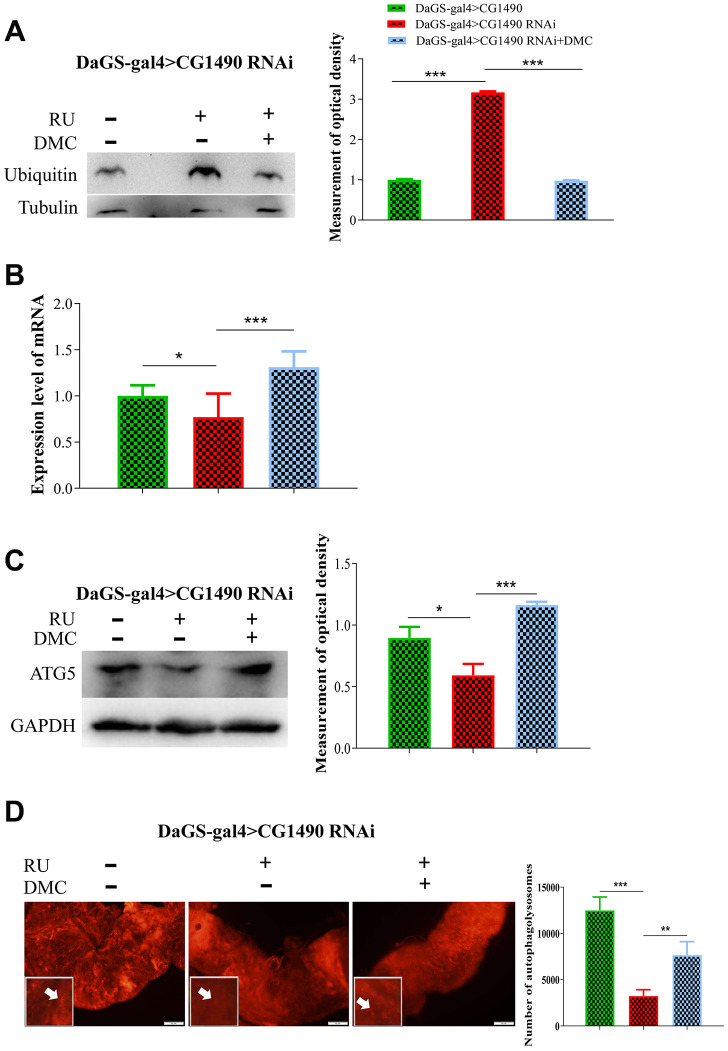
**Effect of DMC addition on protein degradation in *dusp7*-knockdown *Drosophila*.** (**A**) Expression level of ubiquitin protein (*** *p*<0.001). (**B**) Expression level of autophagy-associated gene (* *p*<0.05; ***p<0.001). (**C**) Expression level of autophagy-associated protein (* *p*<0.05; *** *p*<0.001). (**D**) Number of autolysosomes (** *p*<0.01; *** *p*<0.001; white arrow points to autophagosome). (>: hybridization; + means adding corresponding ingredients; - means no adding corresponding ingredients).

To test the effect of DMC on the protein degradation pathway, we investigated changes in the levels of protein ubiquitination, ubiquitin, autophagy, and *atg5* mRNA in *Drosophila*. We found that *dusp7*-knockdown flies had significantly increased levels of protein ubiquitination and ubiquitin protein, but the addition of DMC decreased the level of ubiquitin protein (Figure 8A). Knockdown of *dusp7* significantly decreased the protein and mRNA expression levels of *atg5*, but the addition of DMC increased these levels (Figure 8B, 8C). Additionally, DMC could increase autolysosomes in the midgut epithelial cells (Figure 8D). The results suggest that DMC can enhance autophagy and ubiquitination.

## DISCUSSION

USP7 is an evolutionarily conserved deubiquitinase with high homology between different species [31]. In our study, we found that dUSP7 is necessary to maintain the normal fly lifespan, and flies knocked down for *dusp7* had significantly shortened lifespans and a reduced ability to respond to starvation, oxidative stress and heat stress. In addition, we found that USP7 regulates aging in relation to the autophagy and ubiquitin signaling pathways. Moreover, DMC can partially restore the shortened lifespan and relative aberrant phenotypes caused by *dusp7* knockdown.

The dUSP7 homolog MATH-33 has been shown to regulate lifespan in nematodes by deubiquitinating DAF16 [24]. Our results showed that dUSP7 can also regulate the lifespan of *Drosophila*. When systemic or gut-specific *dusp7* expression in *Drosophila* was knocked down, the lifespan was significantly decreased compared with control groups. This result is consistent with the phenotype of mutant *math-33* in nematodes. With aging, the body's stress tolerance, intestinal function and muscle capacity decline [30, 32]. We found that the systemic knockdown of *dusp7* gave rise to more accumulated aging characteristics than gut-specific *dusp7* knockdown. This suggests that *dusp7* is expressed systemically in *Drosophila*. When knockdown occurred only in the intestine, *dusp7* could also work in other parts of the body, and it could continue to play a compensatory role. Thus, the aging symptoms of *Drosophila* were not as serious under gut-specific *dusp7* knockdown compared to systemic knockdown.

Two pathways have been shown to regulate proteostasis that have been proven to be associated with aging. The first is the ubiquitin-proteasome pathway, which is mainly involved in the regulation of short-lived proteins and regulatory proteins [33]. The second is the autophagic lysosomal pathway, which is mainly involved in the regulation of long-lived proteins [34]. The two pathways work together to keep proteins relatively stable in the body [13]. In our study, when *dusp7* was knocked down, the level of ubiquitination was significantly increased compared with the control. These results indicate that the protein homeostasis is disrupted and the ubiquitinated proteins are increased due to *dusp7* knockdown. Thomas et al. found that MATH-33 removes ubiquitin moieties from DAF-16 to promote DAF-16 stability in response to decreased IIS in *C. elegans* [24]. The human USP7 stabilizes GATA1 by removing K48-linked polyubiquitin [35]. In response to DNA damage, BRE/BRCC45 recruits USP7 to regulate CDC25A stability [36]. These reports demonstrate the significance of USP7 in maintaining protein homeostasis. We also found that the level of autophagy was significantly reduced compared with control. We observed that knockdown of *dusp7* reduces the ability of flies to cope with stress such as starvation and oxidative damage. Therefore, knockdown of *dusp7* could lead to loss of proteostasis in *Drosophila*, possibly leading to a reduced lifespan.

DMC, a celecoxib derivative that has lost the ability to inhibit cyclooxygenase-2 (COX-2), does not cause the side effects of celecoxib; hence, it is used as a celecoxib replacement [28, 29, 37]. It has been reported that DMC can extend the lifespan of wild-type *Drosophila* by activating *foxo*, which is a key factor of the insulin-signaling pathway [30]. However, no reports have yet shown that DMC can restore the lifespan reduction caused by losing protein homeostasis. In our study, we found that DMC could rescue the shortened lifespan of *Drosophila* caused by *dusp7* knockdown. The protein and mRNA expression levels of *atg5* were significantly increased compared with the knockdown group that did not receive DMC, suggesting that the addition of DMC could enhance autophagy. Furthermore, we found that the addition of DMC not only enhances autophagy but also reduces the levels of ubiquitination and ubiquitin protein that are abnormally elevated in *dusp7*-knockdown *Drosophila*. Therefore, we suggest that DMC possibly regulates proteostasis to extend the lifespan of *dusp7*-knockdown *Drosophila*. DMC can rescue parts of the senescence traits of *Drosophila*, but it has no effect on the antioxidation ability. This indicates that DMC may extend the lifespan independent from the increase of antioxidant capacity.

In conclusion, dUSP7 is an essential component for maintaining the normal lifespan of *Drosophila*. Knockdown of *dusp7* could significantly reduce the lifespan, lessen the climbing ability, reduce the stress tolerance and accelerate the loss of intestinal barrier integrity. At the same time, the level of ubiquitin increased and the level of autophagy decreased in *Drosophila*. Finally, the protein homeostasis balance in the body was upset. The insulin signaling pathway inhibitor DMC could partially rescue the most aberrant phenotypes, suggesting that *dusp7* maintains the normal lifespan and partially depends on the insulin-signaling pathway in *Drosophila*. Due to the high conservation of the deubiquitinase dUSP7 across different species, it is expected that the regulatory pathway in aging also could be evolutionarily conserved. These results could provide insights regarding the role of human USP7 in aging and the potential development of targeted drugs.

## MATERIALS AND METHODS

### Fly stocks and feeding

*Da^GS^-gal4*, *5966^GS^-gal4*, *usp7*-RNAi (CG1490, BL34708), and *W^1118^* were obtained from the Bloomington *Drosophila* Stock Center (http://flystocks.bio.indiana.edu), *usp7*-overexpression line (kindly gifted by Professor Zhou). The *Drosophila* were fed with either yeast medium or amino acid medium [38, 39]. The resulting hybrid, *Da^GS^-CG1490 OE* (Over Expression), *Da^GS^-CG1490 RNAi*, *5966^GS^-W1118*, and *5966^GS^-CG1490 RNAi*, was used in experiment.

### Lifespan curve analysis

The survival time was recorded for the selected *Drosophila*. During the experiment, the culture medium of *Drosophila* was replaced every Monday, Wednesday and Friday until the experimental *Drosophila* had all died, and a survival curve was constructed according to the *Drosophila* survival data. Statistical significance analysis was performed using the log-rank test. More than two independent experiments were carried out for most of the lifespan assays.

### Intestinal integrity test of *Drosophila*

Blue dye No. 1 was added to the culture medium for the intestinal integrity tests. When the *Drosophila* were maintained on the dyed medium for 9 hours, if the intestinal function was damaged, the dye entered the body through the intestine, making the whole abdomen and even the whole body blue. At this point, the *Drosophila* are termed "smurfs". In contrast, if the intestinal tract function was intact, the *Drosophila* showed no change.

### *Drosophila* stress index

Each group of experiments required 200 flies that had been fed for 10 days. For the H_2_O_2_ and paraquat assays, *Drosophila* were fed 20 mM paraquat or 5% H_2_O_2_ diluted in a 5% glucose solution supplied on filter paper. In the starvation test, the *Drosophila* were placed in a tube containing 1.5% agarose to provide only moisture. The number of deaths was recorded every four hours in the above experiments. For the heat assay, *Drosophila* were placed in empty tubes at 39° C, and deaths were recorded every hour.

### *Drosophila* climbing ability

A *Drosophila* was placed in a tube, which was gently shaken so that the fruit fly went into the bottom of the tube. The *Drosophila* climbed freely for 45 s and was then photographed. The assay was repeated three times with independent groups of *Drosophila*. The speed was calculated by the formula: PI=(ntot+ntop-nbot)/2/ntot (ntot indicates all *Drosophila*, ntop indicates the *Drosophila* at the top of the tube, nbot indicates the *Drosophila* at the bottom of the tube, and PI stands for climbing ability).

### Western blots

Fruit fly total protein was extracted with a Cell Total Protein Extraction Kit (Sangon Biotech C510003). Aliquots of 40-100 μg protein extract were separated by gel electrophoresis. The proteins were then transferred to polyvinylidene fluoride membranes. The primary antibodies used were as follows: ATG-5 antibody (NOVUS NB110-53818SS), UB antibody (Cell Signaling Technology 3936S), α-tubulin antibody (Sigma T9026), and GAPDH antibody (Servicebio GB11002). All western blot experiments were repeated at least twice.

### qRT-PCR

*Drosophila* cultured for 10 days were frozen on ice, and total RNA was extracted by the TRIzol method and converted into cDNA. The real-time PCR program was performed by the SYBR Green method using the following primers: rp49-F: AGATCGTGAAGAAGCGCACCAAG; rp49-R: CACCAGGAACTTCTTGAATCCGG. atg5-F: TAGGCATATGCTTCCAGGCG; atg5-R: CACAGCTCCATCCTGGTGTT. dusp7-F: GTGAGAATACCTTGGCGGACTACG; dusp7-R: GCGGCAACTGGAGACCACATC. The relative expression levels of the target genes were normalized to the expression level of the housekeep gene *rp49*, and all of the experiments were repeated three times.

### Autolysosome staining analysis

The experimental *Drosophila* were cultured for 20 days, and the guts were extracted and stained with 1 μM LysoTracker DS Red DND-99 (Invitrogen, Molecular Probes) for 3 minutes and then washed 3 times with PBS before mounting and performing microscopy.

### Quantification and statistical analysis

The measurements represent the mean of at least three biological replicates in all graphs, and error bars represent the standard deviation. As appropriate, two-tailed Student’s t-test or one-way ANOVA with post hoc Dunnett’s test was used to calculate significance. All statistical analyses were carried out using GraphPad Prism 6.00 software. For all tests, *p*<0.05 was considered to be statistically significant. Asterisks reflecting the calculated *p*-values are shown above each measurement, and ns indicates that differences between measurements were not statistically significant. No collected data were excluded from any experimental or statistical analysis. Tests for normality or outliers were not performed.

## Supplementary Material

Supplementary Figure 1
