# Supplementary material for: Deubiquitinase USP7 regulates Drosophila aging through ubiquitination and autophagy
Source: Aging (Albany NY). 2020 Nov 20;12(22):23082–95. doi: 10.18632/aging.104067 (PMC7746378; doi:10.18632/aging.104067)
Supplement: Supplementary Figure 1 [file aging-12-104067-s001..pdf]

SUPPLEMENTARY FIGURE

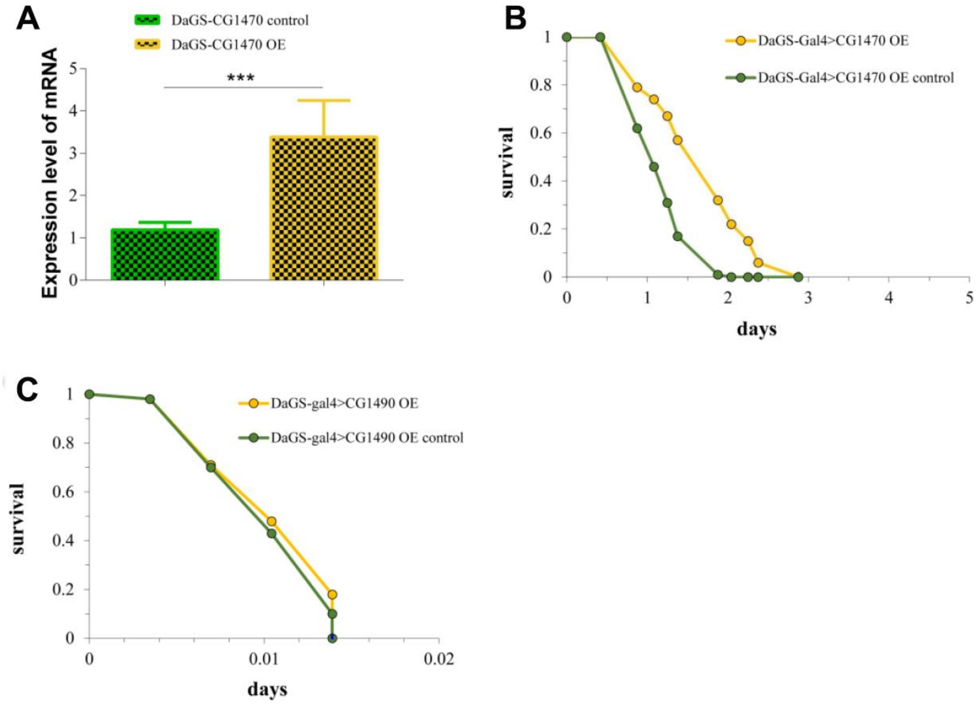

Supplementary Figure 1. Effect of *dusp7* overexpression on *Drosophila* stress tolerance. (**A**) Expression level of *dusp7* mRNA in *dusp7* overexpression flies (*** $p<0.001$). (**B**) Effect of *dusp7* overexpression on Drosophila tolerance to paraquat. (**C**) Effect of *dusp7* overexpression on Drosophila tolerance to heat stimulation.
